# SALL4 activates PI3K/AKT signaling pathway through targeting PTEN, thus facilitating migration, invasion and proliferation of hepatocellular carcinoma cells

**DOI:** 10.18632/aging.204446

**Published:** 2022-12-26

**Authors:** Zhipeng Tang, Pei Zhao, Wanxing Zhang, Qian Zhang, Ming Zhao, He Tan

**Affiliations:** 1 Department of Laboratory, Hebei General Hospital, Shijiazhuang, Hebei 050017, China; 2 Department of Hepatobiliary Surgery, Hebei General Hospital, Shijiazhuang, Hebei 050017, China; 3 Department of Gastrointestinal Surgery, Beijing Tsinghua Changgung Hospital, Beijing 102218, China

**Keywords:** SALL4, hepatocellular carcinoma cells, migration, invasion, proliferation

## Abstract

This study aims to explore the specific mechanisms of SALL4 on the migration, invasion and proliferation of HCC. HepG2 and SMMC-7721 cells were transfected with SALL4 NC, mimics and inhibitors. The proliferation capability and cell cycle progression of HCC cells were detected through CCK8 assay and flow cytometry, and their migration and invasion capabilities were detected by wound healing assay and Transwell assay. In SALL4 inhibitor NC group and SALL4 inhibitor group, the PTEN inhibitor SF1670 was added, and the expression levels of PI3K/AKT, migration, invasion and proliferation-related proteins were detected by Western blotting. Results showed that after up-regulation of SALL4, the migration distance of HCC cells increased, the numbers of migrated cells and the number of colonies formed significantly rosed, and there were fewer cells in G1 phase but significantly more cells in S phase, thereby down-regulation of SALL4, the opposite results. The results of Western blotting revealed that after SF1670, the specific PTEN inhibitor was added in SALL4 inhibitor group and SALL4 inhibitor NC group, the protein expression of PTEN in HCC cells significantly declined, while the protein expressions of p-PI3K, p-AKT, MMP2, MMP9, CyclinD, CyclinA1, PCNA and P62 significantly rose. In conclusion, SALL4 activates the PI3K/AKT signaling pathway through targeting PTEN, thereby facilitating the migration, invasion and proliferation of HCC cells.

## INTRODUCTION

As one of the most common malignancies in clinic [1], primary liver cancer accounted for 47% of the new cases of malignancies (ranking 7th) and 82% of deaths (ranking 2nd), based on the global data of 36 malignancies released in 2018 [2]. In terms of pathological type, approximately 85–90% of primary liver cancer belongs to hepatocellular carcinoma (HCC) [3]. Currently, early surgery is the most effective treatment method for HCC [4]. The 5-year survival rate of patients receiving early diagnosis and treatment is above 70% that is much higher compared with that of patients in late stage (less than 16%) [5]. However, due to insidious onset, most patients suffering from HCC are in the mid or late stage when they come to hospitals for diagnosis, and then they miss the best opportunity for surgery [6]. Therefore, it is of great significance to seek novel biomarkers that are sensitive and highly specific to HCC, as well as new targets for tumor gene therapy.

Human Sal-like 4 (SALL4), a highly important player in the early and mid-stage of fetal liver development, becomes silenced in adults’ liver as one of the few embryonic stem cell (ESC) genes establishing contact with cancer cells [7, 8]. It has been discovered that the zinc finger protein encoded by SALL4 has diagnostic value as a transcription factor in a variety of solid tumors, which makes it a potential tumor marker for HCC [9]. Studies have proved that the phosphatase and tensin homolog deleted on chromosome ten (PTEN)/protein kinase B (AKT) pathway is involved in the induction of HCC by SALL4 [10]. In addition, it was also reported that the silencing of PTEN expression induces the increase of p-AKT level and activation of PI3K/AKT signaling pathway, thereby accelerating the risks of tumorigenesis [11]. However, whether SALL4 can activate PI3K/AKT signaling pathway via inhibiting the expression of PTEN to promote the occurrence and development of HCC has not been confirmed yet.

Therefore, in this present study, the effects of SALL4 on the migration, invasion and proliferation of human HCC HepG2 and SMMC-7721 cell lines were detected, and the possible mechanism was analyzed as well, aiming to lay a foundation for the research on potential biological targets of HCC.

## MATERIALS AND METHODS

### Cell culture and treatment

HepG2 and SMMC-7721 cells were cultured in DMEM with 10% fetal bovine serum (FBS) and 1% double antibodies (penicillin/streptomycin) in a 5% CO_2_ incubator at 37°C. The cells were inoculated in a 6-well plate, and transfected with SALL4 mimic NC, SALL4 mimic, SALL4 inhibitor NC and SALL4 inhibitor, respectively, using the cationic lipid Lipofectamine 2000 upon adherence. They were divided into SALL4 mimic NC group, SALL4 mimic group, SALL4 inhibitor NC group and SALL4 inhibitor group. At 48 h after transfection, the cells were collected and the transfection efficiency was assessed by quantitative polymerase chain reaction (qPCR).

### Detection of cell cycle

At 24 h after culture, the cells were digested, collected, resuspended in 75% ethanol diluted with PBS, and fixed for more than 24 h. Then they were centrifuged and washed with pre-cooled RNase-containing PBS, followed by propidium iodide (PI) staining and incubation in a constant temperature incubator at 37°C for 30 min. Finally, flow cytometry was performed and the results were analyzed.

### Wound healing assay

Cell migration capability was detected by wound healing assay. 1 × 10^6^ cells were inoculated into each well of a 6-well plate. Upon reaching 90% confluence, a wound was created in the central area of single-layer cells using a 10 μL pipette tip. After the medium was discarded, the cells were washed twice with PBS, and added with serum-free DMEM in each well. The images of the migration area were captured under a light microscope at 0 h, 12 h and 24 h, respectively, and the migration distance was measured with ImageJ software.

### Transwell assay

Migration assay: this experiments were carried on in 24-well trans wells uncoated with matrigel (8 μm pore size, corning, USA), 200 μL of serum-free cell suspension was inoculated into the upper Transwell chamber, cultured for 48 h, fixed with paraformaldehyde and stained with crystal violet. Finally, it was observed and counted under a microscope. Invasion assay: this study was performed by using the 24-transwells and pre-coated with matrigel (falcon 354, 480; BD biosciences, USA). The upper Transwell chamber was coated with Matrigel, and the remaining operation was the same as that in migration assay.

### Western blotting

The total protein was extracted from HCC cells in each group, separated by sodium dodecyl sulfate polyacrylamide gel electrophoresis (SDS-PAGE), transferred onto a membrane, mounted and incubated with primary antibodies at 4°C overnight. The next day, it was incubated again with corresponding secondary antibodies for 2 h, the color was developed using developing solution, and the image was exposed and collected.

### Establishment of tumor-bearing nude mice

4 weeks old male BALB/c nude mice were used to establishment the tumor bearing model, and these mice were randomly divided into 4 groups and *n* = 6/group, and all mice were maintained according with institutional animal ethics. SMMC 7721 cells transfected with SALL4 mimic-NC, SALL4-mimic, SALL4 inhibitor-NC, SALL4-inhibitor were collected and 2 × 10^6^ of SMMC 7721 cells were subcutaneously injected into each nude mice, and after 6 weeks of SMMC 7721 bearing into nude mice, the tumors were collected. And moreover, all the process and protocol were approved by the animal use and care committee of Hebei general hospital.

### Statistical analysis

SPSS 20.00 was used for statistical analysis. Measurement data were expressed as $\left(\bar{\text{χ}}\pm \text{s}\right)$, and compared by independent-samples *t* test between two groups, and by one-way analysis of variance among groups. *P* < 0.05 was considered statistically significant.

## RESULTS

### Transfection efficiency of SALL4

**Figure 1:**
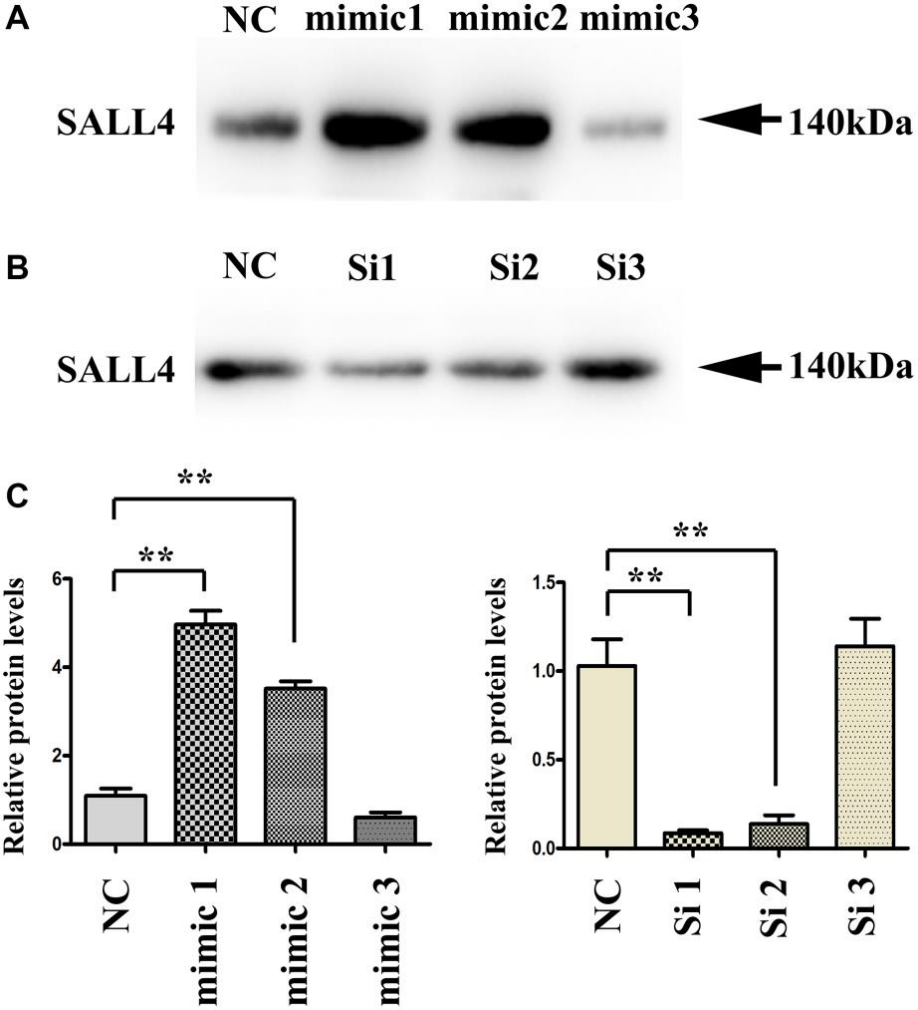
**Transfection efficiency of SALL4 in hepatoma cell lines.** (**A** and **B**) The protein bands after transfection with SALL4 mimic 1, 2, 3 and SALL4 Si 1, 2, 3. (**C**) The protein expression levels of SALL4 after transfection with SALL4 mimic 1, 2, 3 and SALL4 Si 1, 2, 3. *n* = 3/group, *t* test between two groups, and by one-way analysis of variance among groups. ^*^p < 0.05, ^**^p < 0.01 vs. NC group.

The results of western blot showed that the SALL4 protein level in HepG2 and SMMC-7721 cells significantly rose in SALL4 mimic (mimic 1,2,3) group compared with that in SALL4 mimic NC group, while it significantly declined in SALL4 inhibitor (siRNA1,2,3) group compared with that in SALL4 inhibitor NC group, data were shown in Figure 1A–1C.

### SALL4 enhanced proliferation of HepG2 and SMMC-7721 cells

**Figure 2:**
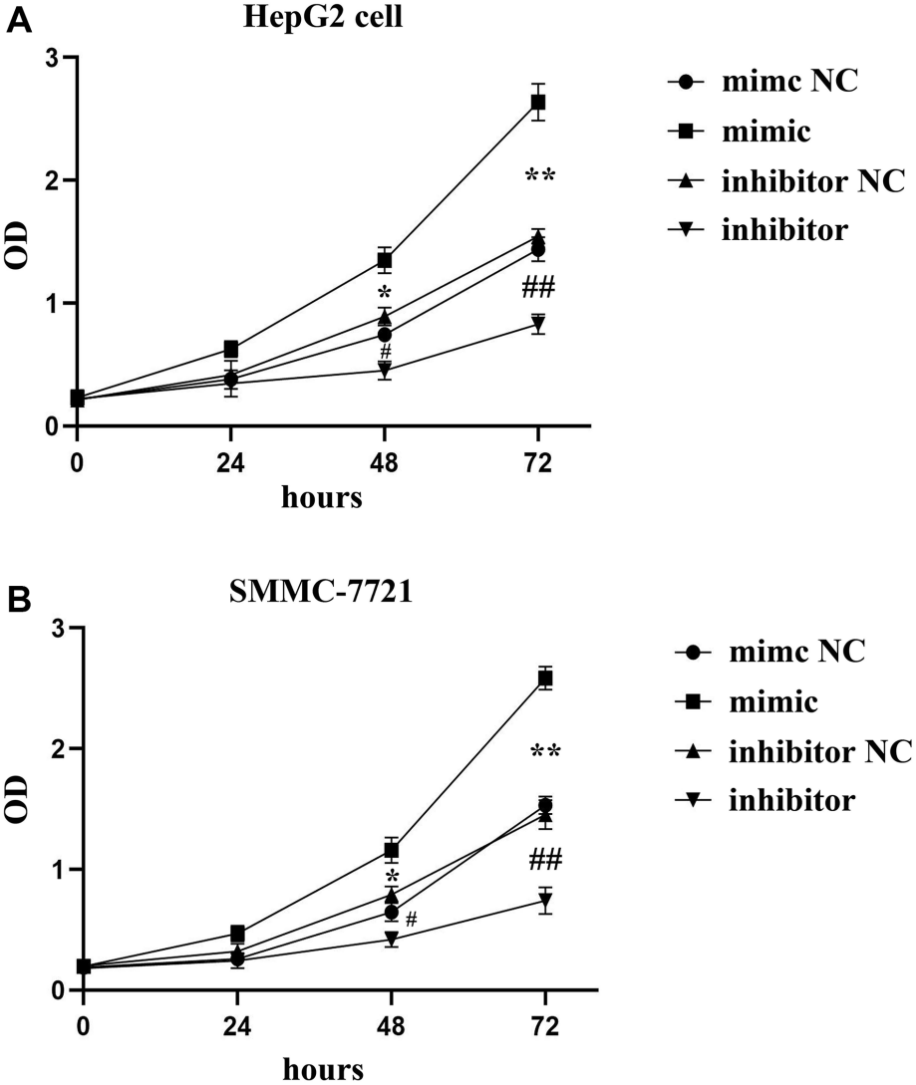
**The roles of SALL4 on the proliferation of hepatoma tested by CCK8 assay.** SALL4 mimic increased the OD value in both HepG2 (**A**) and SMMC-7721 cells (**B**); and SALL4 inhibited the OD value in both HepG2 (**A**) and SMMC-7721 cells (**B**) *n* = 3/group, *t* test between two groups, and by one-way analysis of variance among groups. ^*^p < 0.05, ^**^p < 0.01 mimic vs. mimic NC group, ^#^*p* < 0.05, ^##^*p* < 0.01 inhibitor vs. inhibitor NC group.

The results of CCK8 assay showed that at 48 and 72 h, the OD value was higher in SALL4 mimic group than that in SALL4 mimic NC group, while it was significantly lower in SALL4 inhibitor group than that in SALL4 inhibitor NC group, suggesting that SALL4 enhances the proliferation of HepG2 and SMMC-7721 cells. The OD value were positive correlated with proliferation, thereby, the SALL4 could promoted the hepatoma cells’ hyperplasia and proliferation, and the data were shown in Figure 2A and 2B.

### SALL4 accelerated the cell cycle progression of HepG2 and SMMC-7721 cells

**Figure 3:**
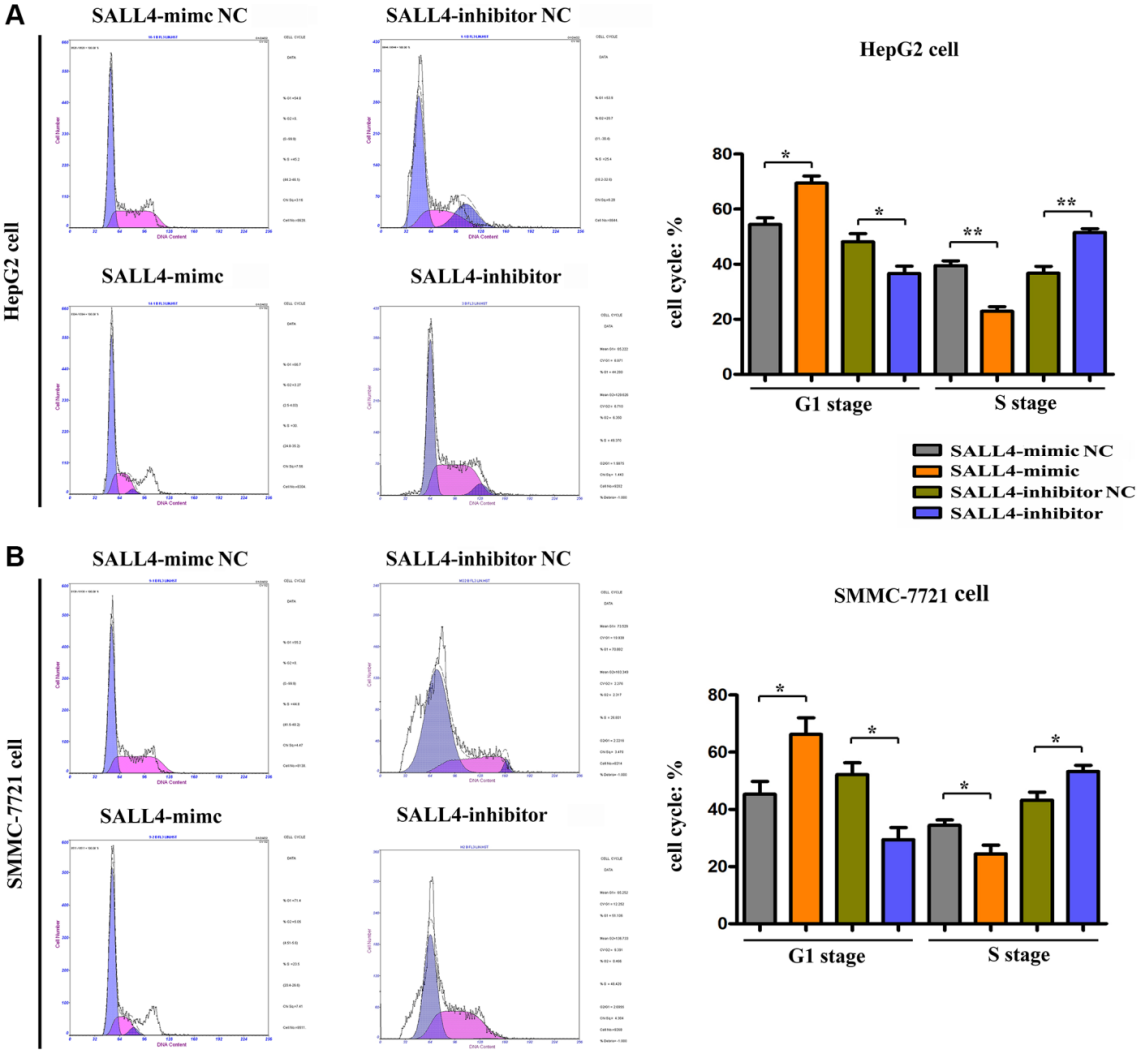
**The effects of SALL4 on the proliferation of hepatic cancers tested by flow cytometry.** SALL4 mimic inhibited the cellular ratio in G1 stage and increased the cellular ratio of S stage in both HepG2 (**A**) and SMMC-7721 cells (**B**); SALL4 inhibitor suppressed the ratio of G1 stage and increased the ratio of S stage in both HepG2 (**A**) and SMMC-7721 cells. *n* = 3/group, *t* test between two groups, and by one-way analysis of variance among groups. ^*^p < 0.05, ^**^p < 0.01 vs. NC group.

The effect of SALL4 on the cell cycle of HepG2 and SMMC-7721 cells was detected via flow cytometry. It was found that after up-regulation of SALL4 of HepG2, SMMC 7721 cells infected with SALL4 mimic resulted into the increased G1 phage cell population, and consistent with decreased cell population of S phage. After down-regulation of SALL4 in HepG2, SMMC 7721 cells transfected with SALL4 inhibitor, the opposite results were obtained as the raised cell population of S phage and suppressed G1 phage. At the same time, the expression changes of cell cycle-related proteins in HepG2 and SMMC-7721 cells were further detected by Western blotting. The results showed that compared with those in SALL4 mimic NC group, the protein expression levels of CyclinD, CyclinA1, PCNA and P62 in HepG2 and SMMC-7721 cells in SALL4 mimic group were significantly increased, suggesting that the overexpression of SALL4 can facilitate the proliferation and G1/S transition of tumor cells, and accelerate the cell cycle progression of HepG2 and SMMC-7721 cells, and the results were shown in Figure 3A and 3B. Combining these results above, the cellular progression as well as proliferation of HCC cells were affected by SALL4, and cell cycle and proliferative proteins were raised by SALL4 protein.

### SALL4 promoted the migration and invasion of HepG2 and SMMC-7721 cells

**Figure 4:**
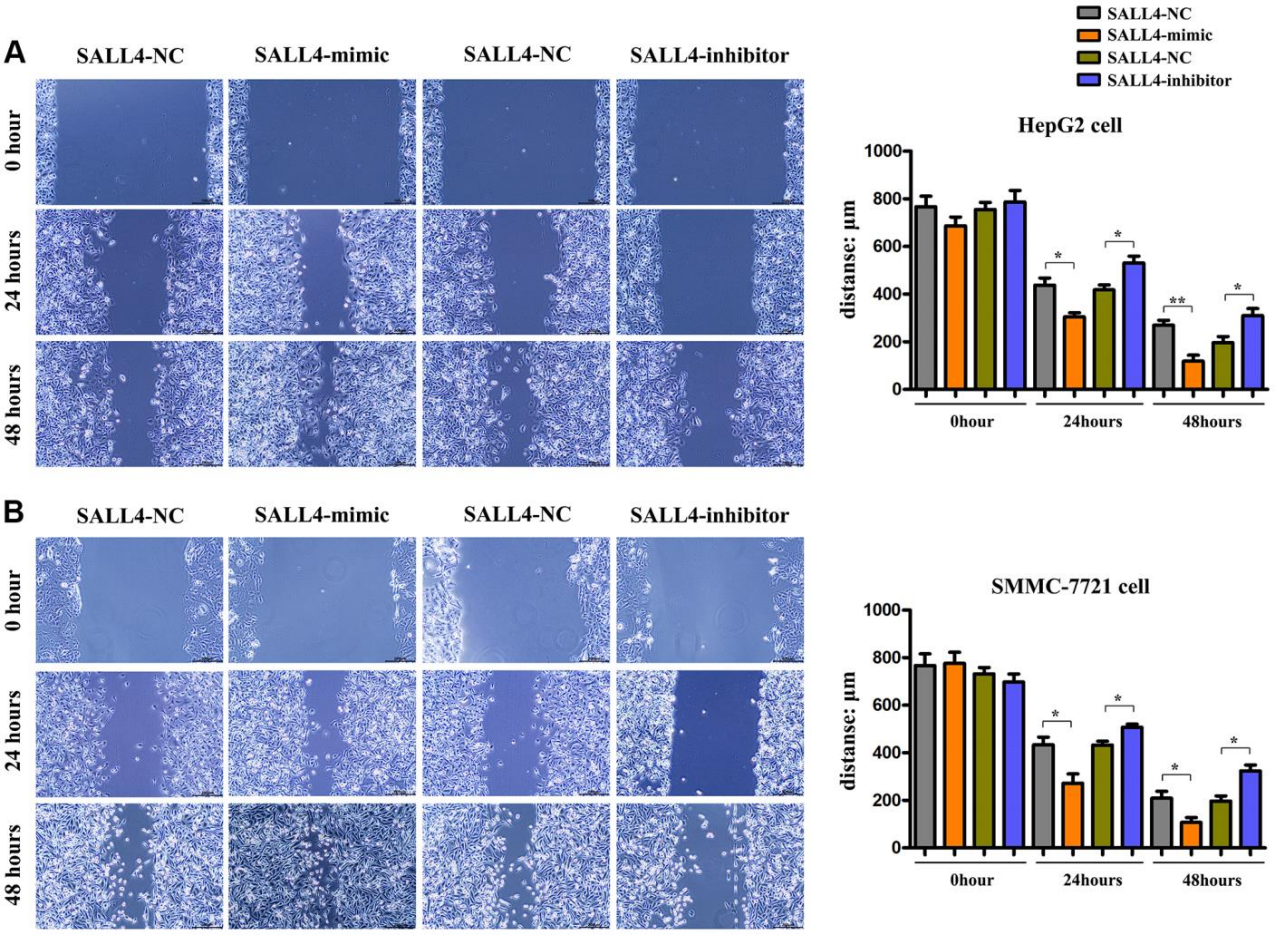
**The effects of SALL4 on the migrated capacity of hepatic cancers.** Distances of cell migration in HepG2 (**A**) and SMMC-7721 (**B**) HCC cells at 0 h, 24 hs and 48 hs after scratch, and the higher expression of SALL4 (mimic) decreased the scratch distance and the inhibited expression of SALL4 (inhibitor) increased the scratch distance after 24 and 48 hs. *n* = 3/group, *t* test between two groups, and by one-way analysis of variance among groups. ^*^p < 0.05, ^**^p < 0.01 vs. NC group.

**Figure 5:**
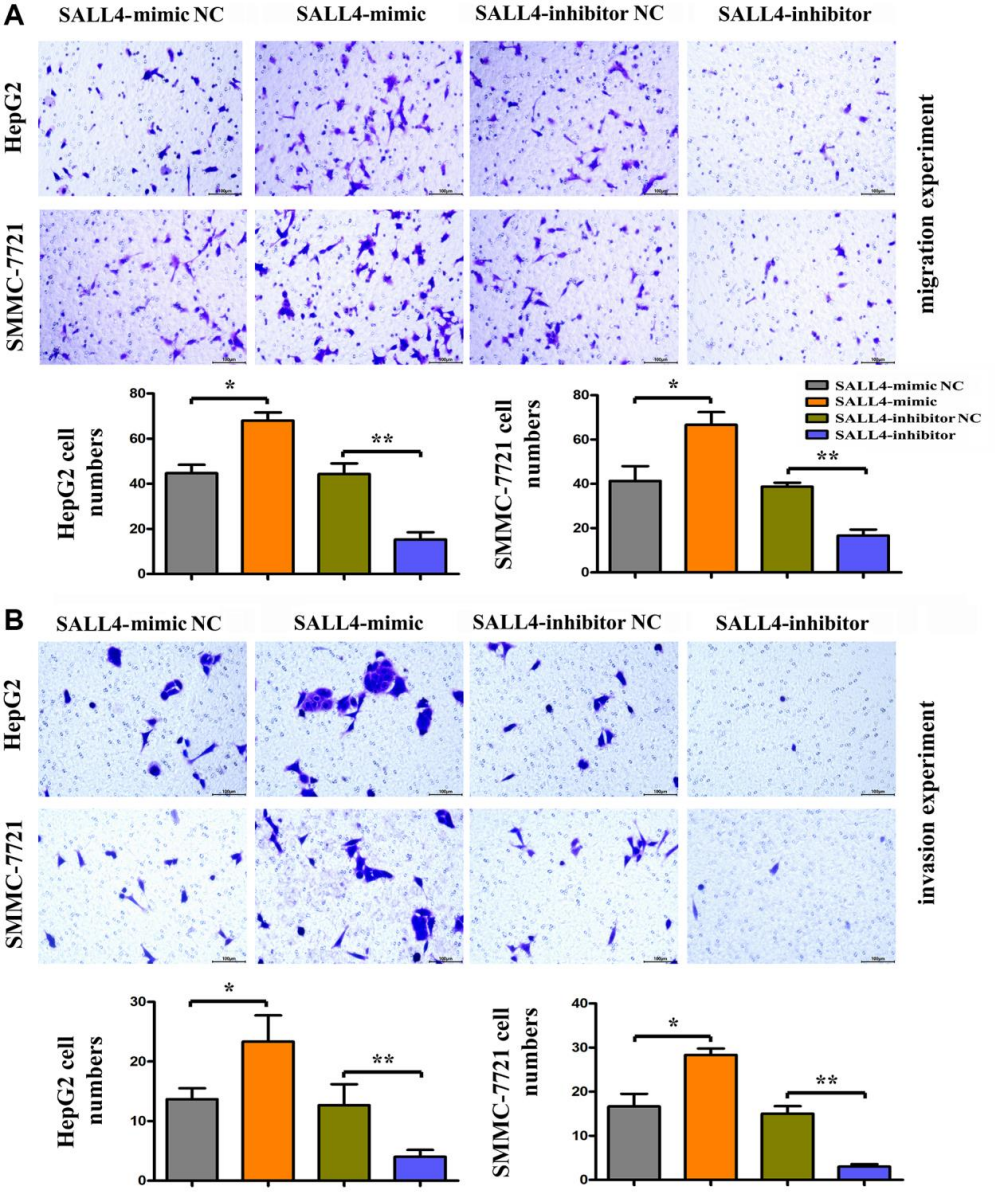
**The roles of SALL4 on the metastatic and invasive ability of hepatoma cells *in vitro*.** (**A**) Migration experiment was performed to detect the number of migration cells in HepG2 and SMMC-7721 HCC cells, the SALL4 mimic increased the migrated numbers and SALL4 inhibitor decreased migrated cell numbers; (**B**) Invasion experiment was used to detect the number of HepG2 and SMMC-7721 HCC cells crossing the basement membrane, the SALL4 mimic increased the numbers of invaded cells and SALL4 inhibitor suppressed the numbers of invaded cells in both HepG2 and SMMC-7721 cells. *n* = 3/group, *t* test between two groups, and by one-way analysis of variance among groups. ^*^p < 0.05, ^**^p < 0.01 vs. NC group.

The results of wound healing assay in HegG2, SMMC 7721 cells revealed that after 24 hours and 48 hours, the wound width or distance in SALL4 mimic group was significantly smaller and closer than that in SALL4 mimic-NC group, indicating that the migrated distance of tumor cells to the center of the wound increased significantly, indicating the SALL4 promoted the migration and invasion’ abilities in HCC cells. Compared with SALL4 inhibitor-NC group, SALL4 inhibitor significantly increased wound width, and these results were shown in Figure 4A and 4B. and consistent with the Transwell results, it was found from Transwell assay that the number of migrated cells and cells passing through the basement membrane was significantly larger in SALL4 mimic group than that in SALL4 mimic-NC group, while it was significantly smaller in SALL4 inhibitor group than that in SALL4 inhibitor NC group and these results were shown in Figure 5A and 5B. The above findings were consistent with the results of western blotting that the protein expressions of MMP2 and MMP9, which are enzymes are able to degrade various components of extracellular matrix (ECM) proteins and promote cancer cell growth and migration, in HepG2 and SMMC-7721 cells were significantly higher in SALL4 mimic group than those in SALL4 mimic NC group. In summary, cell scratch experiments were used to determine the migrated ability of tumor cells, our results about the cell scratch, migration, and invasion experiments identified that, SALL4 accelerated the tumor metastatic capacity.

### SALL4 activated the PI3K/AKT signaling pathway via targeting PTEN to promote the migration, invasion and proliferation of HCC cells

**Figure 6:**
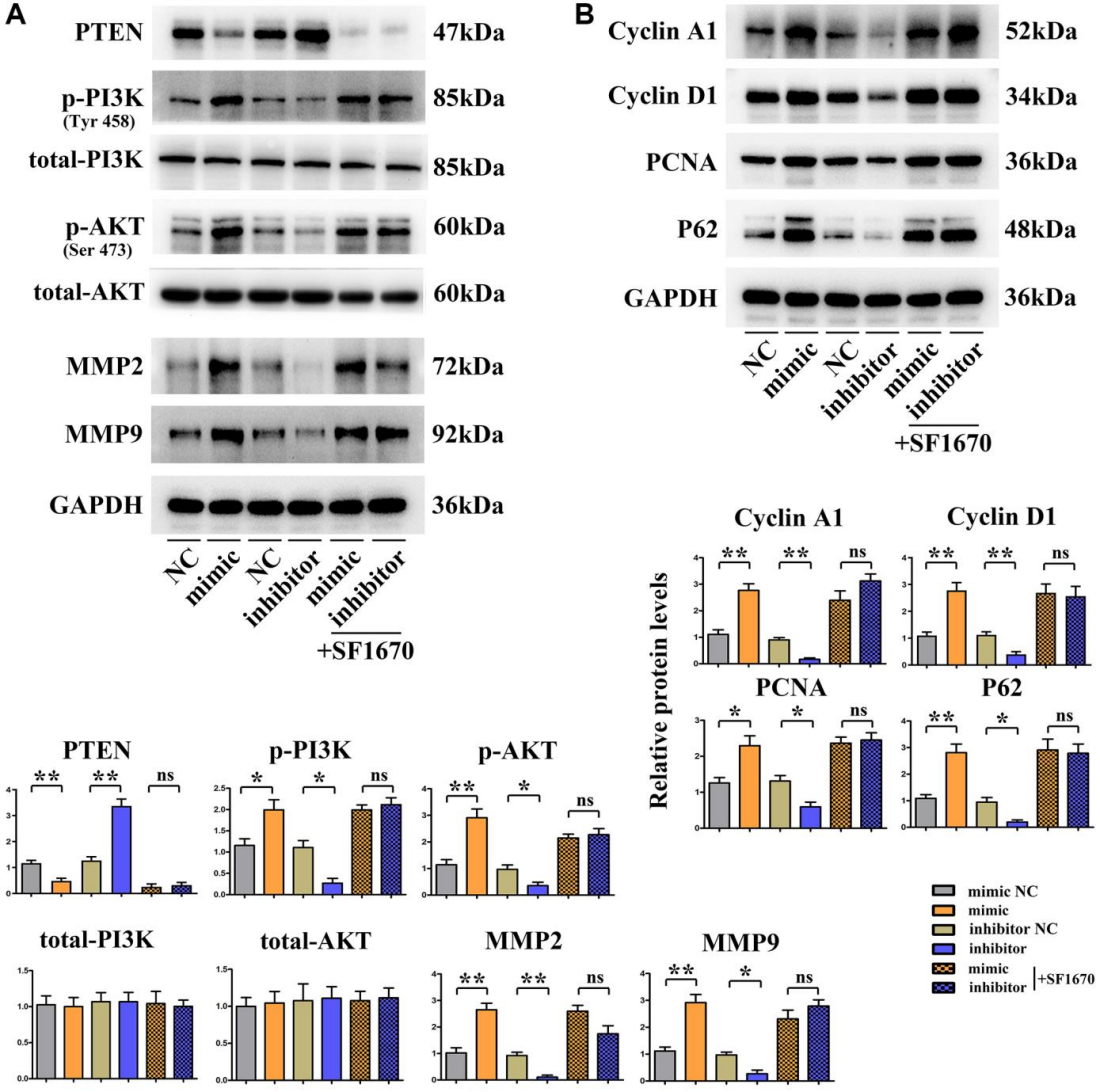
**SALL4 promoted migrated and proliferative ability of hepatic cancer SMMC 7721 cells via modulating PI3K/AKT signals.** (**A**) Changes in protein levels of PTEN, p-PI3K, p-Akt as well as total-PI3K, Akt and downstream proteins: MMP2 and MMP-9 in hepatoma cells, SALL4 mimic increased the phosphorylated-PI3K/AKT and MMP2, -9 consistent with decreased phosphorylated PTEN protein levels and the SALL4 inhibitor showed the opposite effects and SF1670 corrected these phenomenon or effects of SALLU. (**B**) Changes of protein expression levels of CyclinD, Cyclin A1, PCNA and P62 in hepatocellular carcinoma cells. *n* = 3/group, *t* test between two groups, and by one-way analysis of variance among groups. ^*^p < 0.05, ^**^p < 0.01 vs. NC group.

Based on the above results about tumor behavior experiments, it can be seen that SALL4 can promote the migration, invasion and proliferation of HCC cells. The results of Western blotting manifested that SALL4 mimic group had a significantly decreased protein expression of PTEN, the suppressor for PI3K/AKT signals, and significantly increased protein expressions of p-PI3K and p-AKT but not the total-protein levels of PI3K, AKT compared with SALL4 mimic NC group. The contrary trend of the proteins expression were found in SALL4 inhibitor group. At the same time, the specific molecular mechanism of SALL4 affecting the migration, invasion and proliferation of HCC cells was validated by inhibiting the protein expression of PTEN, the SF1670: specific inhibitor for PTEN was used and added into SALL4 inhibitor group and SALL4 mimic group, the protein expression of PTEN in HCC cells significantly declined, while the protein expressions of p-PI3K, p-AKT, MMP2, MMP-9, CyclinD, CyclinA1, PCNA and P62 significantly raised in SMMC-7721 cells. To sum up, SALL4 activates the PI3K/AKT signaling pathway and downstream pro-proliferative proteins through modulating PTEN, thereby facilitating the migration, invasion and proliferation of HCC cells. These results indicated the tumorigenic features of SALL4, at least partly, dependent on the activation of PI3K/AKT signals, and these data were shown in Figure 6.

### SALL4 amplified the progression of HCC cells in nude mice

**Figure 7:**
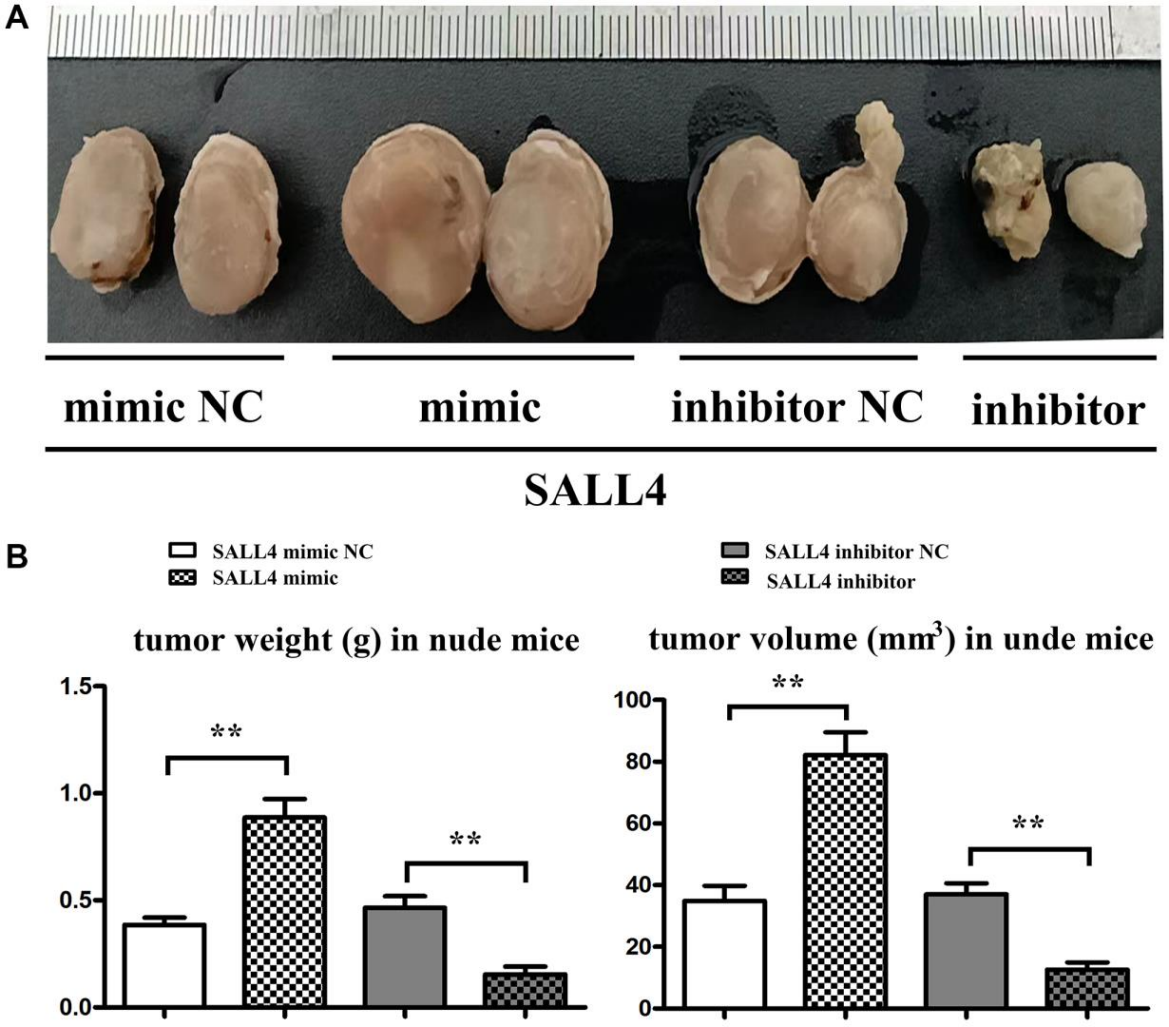
**SALL4 increased the tumor progression in nude mice.** (**A**) SMMC 7721 cells transfected with SALL4 mimic-NC, mimic, SALL4 inhibitor-NC and inhibitor were subcutaneous injected in nude mice; (**B**) Statistical data for tumor weight and volume. ^*^p < 0.05, ^**^p < 0.01 vs. NC group.

SMMC 7721 cells transfected with SALL4 mimic or inhibitor were injected into nude mice and we found SALL4 over expression significantly increased both the weight and volume of tumor and SALL4 inhibition suppressed the cancer growth *in vivo* experiments. These results were consistent with cellular experiments and provided the evidences for the tumorigenic effects of SALL4. And the data were shown in Figure 7.

## DISCUSSION

HCC is one of the common digestive system malignancies [1], with complex mechanism of occurrence and development, and involves multi-gene regulation and changes in signaling pathways [12]. Currently, the diagnosis and assessment of treatment effects and prognosis only depending on serological, clinicopathological and imaging examinations lead to poor diagnosis, treatment and prognosis [2, 13], and the 5-year survival rate of patients with HCC is only about 30% [5]. Therefore, seeking markers that can predict the prognosis and recurrence of HCC and therapeutic targets to inhibit the metastasis is pivotal in exploring HCC [14].

In this study, it was revealed that SALL4 was highly expressed in HCC cells, which was consistent with previous reports that SALL4 was expressed in fetal hepatocytes, rather than adults’ hepatocytes. The expression level of SALL4 gradually declines during the development of liver [15]. Based on this study, the results indicated that SALL4 enhanced the migration, invasion and proliferation of HepG2 and SMMC-7721 cells. After *in vitro* overexpression of SALL4, the number of cells in G1 phase declined, while the number of cells in S phase significantly rose, thereby promoting the G1/S transition and accelerating the cell cycle progression of HCC cells, which was also confirmed by conducting CCK8 assay. CyclinA1 is one kind of cyclin associated with cell malignant transformation [16], and it has been proved that CyclinA1 is highly expressed in various tumor tissues, but not expressed or lowly expressed in normal tissues [17, 18]. CyclinD, an important cell cycle gene, plays an important role in cell cycle regulation [19], whose high expression can promote cell proliferation and differentiation and has close associations with the occurrence and development of tumors [20]. PCNA is closely related to cell proliferation activity, and it has been verified that the proliferation of tumor cells could be suppressed by down-regulating PCNA expression [21]. In this study, the expressions of CyclinA1, CyclinD and PCNA were significantly increased after up-regulating SALL4 in HepG2 and SMMC-7721 cells, which further proved that SALL4 enhanced the proliferation of HepG2 and SMMC-7721 cells. Meanwhile, the migration distance of cells to the center of the wound significantly increased, and the number of migrating cells and cells passing through the basement membrane remarkably rose in SALL4 mimic group compared with those in SALL4 mimic normal contrast (NC) group, which might be related to the changes in protein expression of MMP2 and MMP9. MMP can degrade the extracellular matrix and basement membrane, thus facilitating tumor cell invasion and migration [22]. Moreover, after the up-regulation of SALL4, the protein expressions of MMP2 and MMP9 in HepG2 and SMMC-7721 cells greatly rose, which also further suggested that SALL4 can enhance the migration and invasion of HepG2 and SMMC-7721 cells, and moreover, SALL4 mimic also increased the HCC tumor progression in nude mice experiment, identified the tumorigenicity of SALL4 in hepatic cancer.

To explore the specific mechanism of SALL4 in facilitating the migration, invasion and proliferation of HCC cells, PTEN and PI3K/AKT signaling pathway were studied. According to the results, after the overexpression of SALL4 in HCC cells, the protein expression of PTEN was significantly reduced, but the protein expressions of p-PI3K and p-AKT were remarkably raised, which indicated that SALL4 may activate the PI3K/AKT signaling pathway through targeting PTEN, thereby affecting the biological behavior of HCC cells. It was reported that the silencing of PTEN expression could induce the increase of p-AKT level and activation of PI3K/AKT signaling pathway, thus increasing the risks of tumorigenesis [11]. Therefore, the specific molecular mechanism of SALL4 in affecting the migration, invasion and proliferation of HCC cells was validated by inhibiting the protein expression of PTEN. After SF1670 was added in SALL4 inhibitor group and SALL4 inhibitor NC group, the protein expression of PTEN in HCC cells significantly declined, while the protein expressions of p-PI3K, p-AKT, MMP2, MMP9, CyclinD, CyclinA1, PCNA and P62 obviously rose. In conclusion, SALL4 could activate the PI3K/AKT signaling pathway through targeting PTEN, thus facilitating the migration, invasion and proliferation of HCC cells. Our luciferase reporter assay identified the SALL4 failed to interaction of the promotor of PTEN, and combined with previous studies showed that SALL4 could modulate the miR-188-5p, which suppressed PTEN messenger RNA and protein expression in gastric cancer [23], and moreover SALL4 associates with nucleosome remodeling deacetylase (NuRD) to silence tumor-suppressor genes, such as PTEN [24], thereby SALL4 may affect the expression and activation of PTEN indirectly. In conclusion, we found that SALL4 promoted the progression of hepatic cancer by accelerating the proliferation and invasion and metastasis ability via modulating PTEN, PI3K/AKT signals.
